# Emotion Regulation Failures in Daily Life in Younger and Older Adults

**DOI:** 10.1093/geroni/igaf122.3274

**Published:** 2025-12-31

**Authors:** Blake Ebright-Jones, Jacob Gurera, Derek Isaacowitz

**Affiliations:** Washington University in St. Louis, St. Louis, Missouri, United States; U.S. Center for SafeSport, Denver, Colorado, United States; Washington University in St. Louis, St. Louis, Missouri, United States

## Abstract

Can better emotion regulation abilities explain why older adults report higher levels of emotional well-being? While most research compares age groups on emotion regulation attempts, we considered whether older adults report fewer emotion regulation failures than younger adults. We conducted a daily diary study asking for daily reports every day for 10 days (n = 1036 observations). We asked younger adult participants (n = 79, Mage = 20.6, SDage = 1.92) and older adult participants (n = 75, Mage = 73.2, SDage = 8.47) to identify episodes in which they had attempted to regulate their emotions. For these attempts, participants indicated whether they had succeeded or failed to regulate, and the valence, arousal, and effort associated with their regulation attempt. In predicting emotion regulation failures, we did not find a significant age difference in the likelihood of success vs. failures (OR = 0.70, p = 0.111). Instead, the use of positive-approach regulation tactics (i.e., focusing attention on positive aspects of a situation) was associated with emotion regulation failures for all ages (OR = 2.91, p = 0.001). Among those participants who experienced an emotion regulation failure, older adults were more likely to report positive feelings (β = 0.23, p = .007), higher arousal (β = 1.33, p < .001), and greater effort expenditure in emotion regulation (β = 0.27, p = 0.018). Emotion regulation failures may not differentiate younger and older adults overall but may still provide some clues about how age groups differ in their unfolding emotion regulation behavior.

